# The biological functions of sphingolipids in plant pathogenic fungi

**DOI:** 10.1371/journal.ppat.1011733

**Published:** 2023-11-09

**Authors:** Xue-Ming Zhu, Lin Li, Jian-Dong Bao, Jiao-Yu Wang, Asen Daskalov, Xiao-Hong Liu, Maurizio Del Poeta, Fu-Cheng Lin

**Affiliations:** 1 State Key Laboratory for Managing Biotic and Chemical Treats to the Quality and Safety of Agro-products, Institute of Plant Protection and Microbiology, Zhejiang Academy of Agricultural Sciences, Hangzhou, China; 2 State Key Laboratory of Rice Biology, Institute of Biotechnology, Zhejiang University, Hangzhou, China; 3 Department of Microbiology and Immunology, Stony Brook University, Stony Brook, New York, United States of America; 4 Division of Infectious Diseases, Stony Brook University, Stony Brook, New York, United States of America; 5 Veterans Affairs Medical Center, Northport, New York, United States of America; Rutgers University, UNITED STATES

## Abstract

Sphingolipids are critically significant in a range of biological processes in animals, plants, and fungi. In mammalian cells, they serve as vital components of the plasma membrane (PM) in maintaining its structure, tension, and fluidity. They also play a key role in a wide variety of biological processes, such as intracellular signal transduction, cell polarization, differentiation, and migration. In plants, sphingolipids are important for cell development and for cell response to environmental stresses. In pathogenic fungi, sphingolipids are crucial for the initiation and the development of infection processes afflicting humans. However, our knowledge on the metabolism and function of the sphingolipid metabolic pathway of pathogenic fungi affecting plants is still very limited. In this review, we discuss recent developments on sphingolipid pathways of plant pathogenic fungi, highlighting their uniqueness and similarity with plants and animals. In addition, we discuss recent advances in the research and development of fungal-targeted inhibitors of the sphingolipid pathway, to gain insights on how we can better control the infection process occurring in plants to prevent or/and to treat fungal infections in crops.

## Introduction

Cells have developed numerous mechanisms to sense environmental changes and to generate responses, with the aim of maintaining cellular homeostasis [1]. The plasma membrane (PM) is not merely a cell barrier but rather a dynamic interface that selectively controls the entry and exit of a myriad of small molecules, nutrients, and substances supporting cell survival [2–4]. Moreover, the PM is a scaffolding platform for protein transporters and for signaling proteins that transmit signals from the outside to the inside of the cell, or vice versa, or/and from cell to cell (intercellularly) [5]. In eukaryotes, the PM primarily contains lipids (e.g., phospholipids, sterol lipids, glycolipids, and sphingolipids) [6] and those proteins that are embedded in the lipid bilayer, function as receptors, transporters, channels, or signal transducers [7]. As revealed by several investigations, lipids are not homogenously distributed in the lipid bilayer to simply provide a structural barrier but rather are carefully positioned to support membrane cell signaling or membrane movement or/and invagination [8,9]. For example, in mammalian cells certain sphingolipids and cholesterol do assemble with specific proteins in lipid rafts [10] to promote the invagination and the internalization of various microbial pathogens [11–15]. In fungal cells, certain sphingolipids do assemble at the bud neck to promote growth of the daughter cell and its separation from the mother cell [16]. This suggests that the lipid composition of the PM is not static but is rather dynamic and may change significantly during the cellular life to respond a specific biological need.

In eukaryotic cells, sphingolipids are generated in the endoplasmic reticulum (ER) and are then transported to and embedded in the PM [17–19]. Tens of thousands of sphingolipids and their metabolites have been reported to be present in mammalian cells. In mammalian cells, studies have shown that sphingolipids are involved directly or indirectly in keeping the PM structure intact and in signaling messages through the membranes by contributing to the regulation of membrane fluidity, stability, resistance to various stress, and, eventually, in controlling programmed cell death [20–24]. Of importance, the effect of 2 sphingolipids, sphingomyelin and ceramide, on the melting temperature of the PM is particularly important because small changes of the extracellular temperature can significantly affect the function(s) of mammalian cells. This is well-known in plant cells, as plant sphingolipids are notoriously known to sense change in environmental temperature and to respond accordingly by re-arranging existing or/and by synthesizing and incorporating new lipids in the PM [25]. This suggests that the rise of environmental temperature due to climate change will have a much more severe effect on humans than plants because plants already have specific sensors to cope with temperature change in the environment. Recent research has also suggested that sphingolipids are critical to microbial pathogens, which have evolved a variety of strategies to use sphingolipids for surviving in harsh ambient environments and in infected mammalian and plant tissues [23,26].

In this review, we discuss the biological functions and metabolic pathways of sphingolipids at different stages of cell development and during the infection cycle of plant pathogenic fungi. We also highlight similarities and differences between microbial, plant, and human sphingolipids in the attempt to stimulate the development of novel treatment options to prevent or treat crop diseases. Finally, we hope to give a perspective on the processes governing the impact of climate change and the rise of environmental temperature on microbial and host cellular physiology.

### 1. The de novo synthesis system of sphingolipids

The synthesis of sphingolipid originates in the ER, an intracellular organelle that in addition to synthesis also participates in the processing, packaging, and transport of proteins and lipids [27]. The serine palmitoyltransferase (SPT) enzyme binds to ER membrane orosomucoid proteins Orms/ORMDLs in fungi, plants, and animals [28–30], and catalyzes the condensation of serine and fatty acyl-CoA, yielding 3-ketodihydrosphingosine (3-KDS), the first sphingoid base, also termed long chain sphingoid bases (LCBs). LCBs serve as the basic building blocks of all sphingolipids. SPT acts as the rate-limiting step in the sphingolipid de novo biosynthesis system [31,32], and it is highly conserved in all eukaryotes, whereas downstream enzymes have significantly diverged in various species [17]. In animals, 3-KDS is highly toxic, thus, as soon as it is produced, the 3-ketosphinganine reductase (KDSR) immediately transforms it into dihydrosphingosine (DHS), which is the most abundant LCB in mammalian cells.

In plants, there are more than 1 LCB and the most abundant are 4-hydroxysphinganine (phytosphingosine, t18:0), 4-hydroxysphingosine (t18:1), sphingosine (d18:1), and sphingadienine (d18:2). This difference between mammals and plants is due to a unique LCB biosynthetic pathway that involves LCB delta (4)- and delta (8)-desaturases as well as LCB C4-hydroxylases. This implies that, in plants, ceramides and more complex sphingolipids have different LCB backbones. In humans, DHS is further catalyzed into dihydroceramide (DHCer) through acylation by 6 different ceramide synthases (CerS1-6). The next de novo step is catalyzed by dihydroceramide desaturase (Des1), transforming DHCer into ceramide. In plants, similar CerS are responsible for the synthesis of ceramides, although only 3 CerS have been identified in plants and are divided in 2 classes: the ceramide synthase LOH2 produces ceramides with long chain fatty acids, whereas LOH1 and LOH3 produce ceramides with very long chain fatty acid (VLCFA).

**Fig 1:**
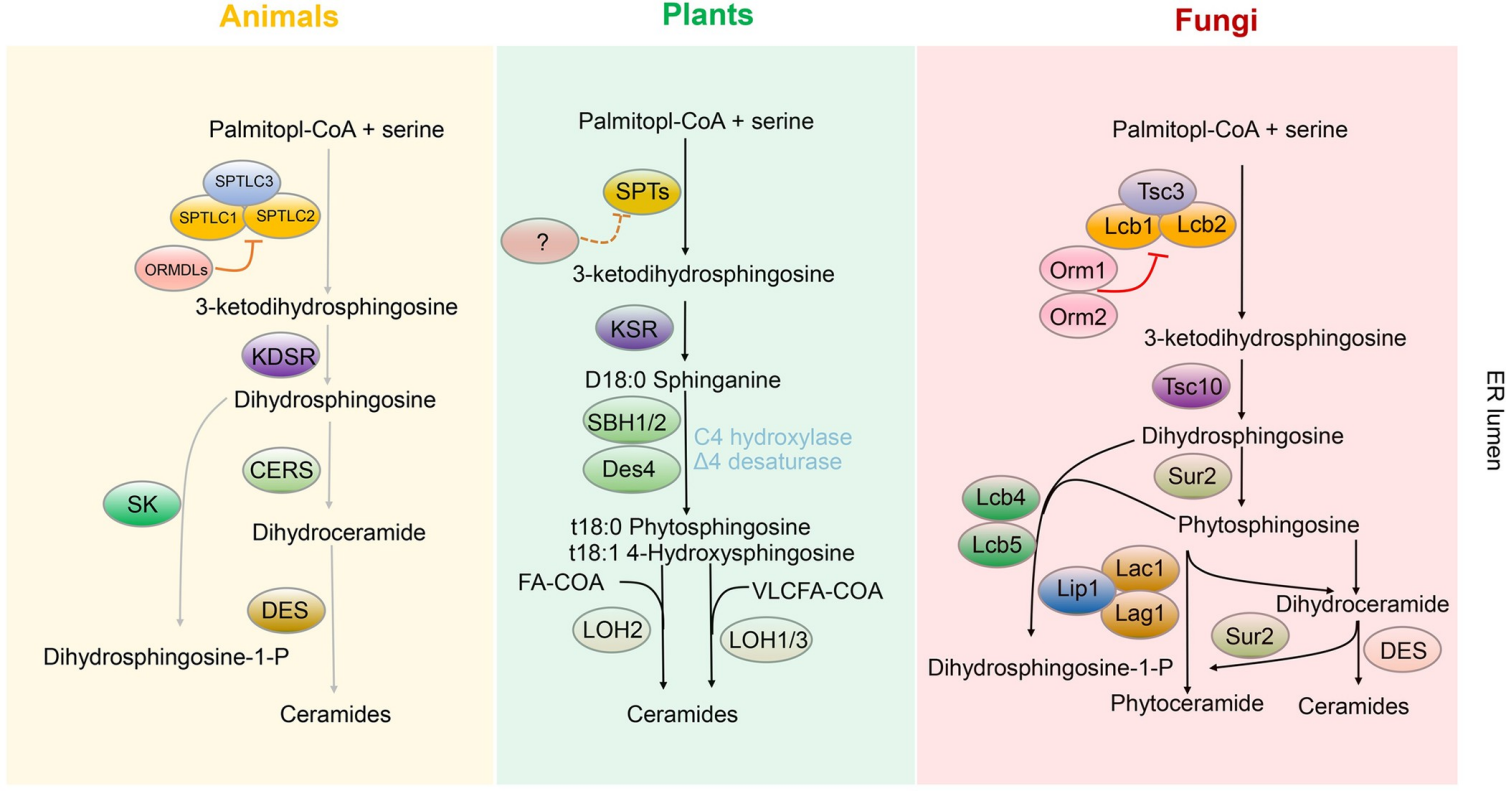
The de novo sphingolipids synthesis pathway in animals, plants, and fungi. Serine and fatty acyl-CoA as raw materials are catalyzed by serine palmitoyl transferase to form 3-KDS, before being transformed into dihydrosphingosine through 3-ketosphinganine reductase. The dihydrosphingosine is further catalyzed to dihydroceramide by ceramidases and then form ceramide by Δ4-desaturase. In animals, the dihydrosphingosine can be phosphorylated to dihydrosphingosine-1-P (S1P) by S1P lyase. In plants and fungi, the dihydrosphingosine can form phytosphingosine and phytoceramide by additional enzymes that introduces a double bond in LCB, which is absent in animals. 3-KDS, 3-ketodihydrosphingosine; LCB, long chain sphingoid base; S1P, sphingosine-1-phosphate; SPT, serine palmitoyltransferase; VLCFA, very long chain fatty acid.

Similarly to animals and plants, fungal LCBs are generated through SPT with palmitoyl-CoA and serine as substrates. LCBs are also highly toxic to fungal cells and thus immediately used to make ceramides and more complex sphingolipids. There are also 3 fungal CerS in fungi, but, in contrast to human or plant CerS, the fungal CerS are capable to attach an enormous variety of fatty acids to their LCBs. Fungi also catalyze DHS into phytosphingosine by C4-hydroxylase Sur2. However, whereas the production of phytosphingosine in mammalian cells is restricted in certain tissues, phytosphingosine production in fungal cells is quite abundant as the corresponding phytoceramides are then preferentially used to make inositol-containing sphingolipids. Interestingly, in fungi pathogenic to plants these inositol-containing sphingolipids are further highly glycosylated [33]. This glycosylation also occurs in fungi pathogenic to humans (e.g., *Cryptococcus neoformans*, *Candida albicans*, or *Aspergillus fumigatus*) but to a much lesser extent. It has been suggested that perhaps this specific glycosylation in fungi pathogenic to plants may help to protect fungal cells from plant defensin(s). It is not known if a similar effect is in place in fungi pathogenic to humans in protecting against human defensin(s) but it might be so because fungal mutants lacking these inositol-containing sphingolipids are highly susceptible to antimicrobial peptides [34]. As the methods for analyzing sphingolipids are improving, we are now beginning to decipher the complexity of their structure and their biological function (Fig 1).

### 2. Structural diversity of complex sphingolipids in fungi, plants, and animals

The PhytoCer or DHCer serves as substrates for the synthesis of complex sphingolipids in the Golgi complex. The complex sphingolipids exert some similarities and uniqueness in animals, plants, and fungi. In animals, ceramide is mainly used to produce sphingomyelin (SM), GlcCer, and GalCer. Fungi do not produce SM but they produce GlcCer and GalCer, although their structure is different. Specifically, fungal GlcCer and GalCer have their LCB backbone further desaturated in position 8 and methylated in position 9 by 2 enzymes (Sld8 and Smt1, respectively) not present in mammalian cells. Sld8 is present in plant cells but not Smt1. Thus, plant GlcCer contains LCBs desaturated in position 8 but not methylated in position 9 [35]. Furthermore, ceramides can be hydrolyzed by ceramidases (CDases) to yield sphingosine, which then can be phosphorylated by sphingosine kinases (SphK) to generate sphingosine-1-phosphate (S1P). S1P is an important signaling molecule in mammalian cells involved in a variety of cellular processes. There are approximately 1,800 reviews online on S1P and its function(s) in various organisms. Plants do produce S1P and phytosphingosine-1-phosphate, whereas fungi almost exclusively produce phytosphingosine-1-phosphate. However, under physiological conditions, the level of phytosphingosine-1-phosphate in fungal cells is very low, almost undetectable, by liquid chromatography–mass spectrometry (LC–MS) analysis.

**Fig 2:**
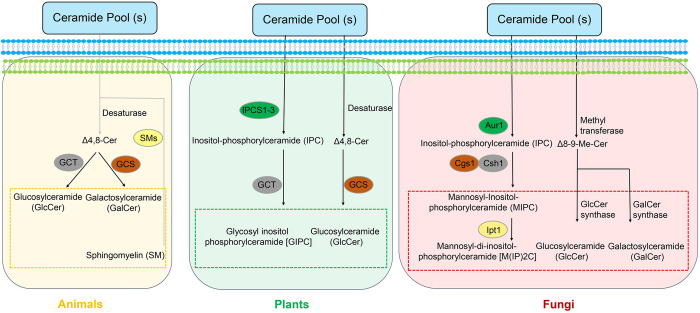
The complex sphingolipid metabolic pathways in Golgi complex. In animals, plants, and fungi, the specific complex sphingolipids are generated in the Golgi by different catabolic enzymes. Ceramide is catalyzed to produce GalCer and GlcCer in animals and plants, whereas GalCer or GlcCer is structurally different and display different abundance in the PM. In addition, in *S*. *cerevisiae* only MIPC was formed while in other pathogenic fungi and plants form not only GIPC but also GlcCer. GIPC, glycosyl inositol phosphoryl ceramide; IPC, inositol-phosphoryl ceramide; PM, plasma membrane.

In plants, PhytoCers are used to produce complex sphingolipids. Firstly, inositol phosphoryl ceramide synthase 1 (Ipc1) transfers an inositol-phosphate group to the C1 hydroxyl of PhytoCer to form inositol-phosphoryl ceramide (IPC) [36]. Then, various glycosyltransferases further attach additional sugars to IPC to generate glycosyl inositol phosphoryl ceramides (GIPCs) [37]. In fungi, PhytoCer is used by Ipc1 (also called Aur1 in yeasts) to produce IPC, one of the most abundant complex sphingolipids in fungi and the most abundant in yeasts. Like in plants, IPC is further processed by various glycosyltransferases to produce GIPCs, MIPC, M(IP)2C, M2IPC, and may be additional complex sphingolipids [23] (Fig 2).

In general, pathogenic fungi have abundant GlcCer, mainly 1 specie, compared to inositol-containing sphingolipids. Plant cells have abundant inositol-containing sphingolipids and a huge variety of GlcCer (approximately 70 species) [38]. Mammalian cells have abundant SM and very little GlcCer variety because in mammals GlcCer represents the building block of more complex sphingolipids, such as cerebrosides and sulfatides [39]. Table 1 lists the sphingolipid synthesis-associated genes and sphingolipid products in animals, plant, and fungi, and provides some insights into the sphingolipid synthetic pathway in plant pathogenic fungi.

**Table 1:** The sphingolipid synthesis-associated genes in animals, fungi, and plants.

Proteins	Animals		Plants		Fungi			
	*Homo sapiens*	Function/phenotype	*Arabidopsis thaliana*	Function/phenotype	*Saccharomyces cerevisiae*	Function/phenotype	*Magnaporthe oryzae*	Function/phenotype
ORM	ORMDL1 ORMDL2 ORMDL3	Negative regulators of SPT complex [17,30]	At5g42000 At1g01230	Contribute to sphingolipid homeostasis and stress responses [40]	Orm1 Orm2	Mediate sphingolipid homeostasis [41,42]	MGG_16259	Not reported
SPT	SPTLC1 SPTLC2 SPTLC3	Catalyzes serine with palmitoyl-CoA to form 3-ketosphinganine [43]	At4g36480 At5g23670 At3g48780	Chimeras of the yeast and arabidopsis LCB2 genes complement the lcb2 null mutant [40]	Lcb1 Lcb2 Tsc3	Catalyzes serine with palmitoyl-CoA to form 3-ketosphinganine [44]	MGG_00864 MGG_05197 MGG_13924	Not reported
KSR	KDSR	Mutations in human cause recessive progressive symmetric erythrokeratoderma [45]	At3g06060 At5g19200	Not reported	TSC10	Catalyzes phytosphingosine synthesis	MGG_00362	Not reported
Δ4-desaturase	DES1	Increase ceramide levels and cell apoptosis [46]	At4g04930	No obvious defects in mull mutant [38]	_	_	MGG_13187	Not reported
C4-hydroxylase	_	_	At1g14290 At1g69640	No obvious defects in single null mutant [47]	SUR2	Catalyzes the conversion of sphinganine to phytosphingosine (PHS) in sphingolipid biosynthesis [48].	MGG_06250	Not reported
Ceramide synthase	CERS	Importance for purkinje cell development in the forebrain and cerebellum [49]	At3g25540 At3g19260 At1g13580	No obvious defects in single null mutant [50]	LAG1 LAC1 Lip1	Involved in synthesis of ceramide, dihydrosphingosine or phytosphingosine [51]	MGG_0309 MGG_05189	Impaire ceramide production and cell growth [51]
Δ8-desaturase	FADS2	As a tumor suppressor in neoplastic disorders [52]	At3g61580	No obvious defects in single null mutant [53]	_	_	_	_
LCB kinase	SK	Generate sphingomyelin [54]	At4g21540	No obvious defects in null mutant [55]	LCB4 LCB5	Phosphorylates dihydrosphingosine to form dihydrosphingosine-1-phosphate [56]	MGG_05147	Not reported
IPCS	_	_	At2g37940	No obvious defects in null mutant [57]	AUR1	Catalyzed phytoceramide to inositolphosphoceramide [43]	MGG_13347	Not reported
Glycosyltransferases	GCS GCT	In response to cardiovascular disease, diabetes, skin disorders, and cancer [49]	GCS GCT	Seedlings are unusually small in null mutant [58]	_	_	MGG_1066 MGG_13744 MGG_15239	Impaire appressoria development [51]
MIPC synthases	_	_	_	_	Cgs1 Chs1	Generate MIPC [43]	_	_
IPT	_	_	_	_	Ipt	Generate M(IP)2C [43]	_	_

Note:—:No homology gene in these species; ORM: orosomucoid proteins; SPT: serine palmitoyltransferase; KSR: 3-ketodihydrosphingosine reductase; IPCS:nositol-phosphoryl ceramide synthase; IPT: inositolphosphotransferase.

### 3. The biological functions of sphingolipids in pathogenic fungi

Research on sphingolipids in pathogenic fungi has intensified over the past few years, mainly because they have been exploited as new, attractive, and unique antifungal targets. In addition, they regulate cellular signaling processes, such as polarized growth and fungal virulence [51,59]. Thus, targeting their function(s) will eventually kill or dramatically effect fungal growth, fungal survival, and fungal virulence. These studies were initiated in the model fungal organism *Saccharomyces cerevisiae*, and then expanded in other fungal pathogens, mostly afflicting humans [22,60–62].

In plant pathogenic fungi, sphingolipids and their derivatives have been confirmed to play a critical role in the regulation of fungal pathogenicity. For example, *Fusarium graminearum* is an ascomycete species causing disastrous *Fusarium* head blight disease on cereal crops worldwide [63]. The gene responsible for GlcCer production in *F*. *graminearum* is *FgGCS1*. Deletion of *FgGCS1* produces a mutant lacking GlcCer (Δ*Fggcs1*). This mutant is still capable to colonize wheat heads and corn silk, but its capacity to propagate in these tissues is significantly reduced compared with to the wild-type strain [64]. Interestingly, the Δ*Fggcs1* was still fully virulent in tomato fruits and in *Arabidopsis thaliana* foliar tissues [65], suggesting that the role of GlcCer in fungi affecting plants is host-dependent. A key feature of plant pathogenic fungi is the presence of 2 sphingolipid C9 methyltransferases, *FgMT1* and *FgMT2*, that, as discussed above, methylate the LCB backbone of ceramide in position 9, only in fungi. Most, if not all, human pathogenic fungi have only 1 gene encoding for the sphingolipid C9 methyltransferase activity. Deletion of the 2 genes mentioned above results in significantly reduced virulence of *F*. *graminearum* [66]. It is not known how this methylation controls virulence but it is possible that the methyl group of GlcCer may have strong hydrophobic interactions with the side chains of other lipids in the bilayer fungal membrane.

That GlcCer is an important virulence lipid was validated by Rittenour and colleagues [67]. They found that the ceramide synthase Bar1 in *F*. *graminearum* controls GlcCer production and hyphal growth and the Bar1 mutant is not pathogenic [67]. These results indicate that, similarly to pathogenic fungi to humans, fungi pathogenic to plants also require GlcCer and particularly methylated GlcCer, suggesting that methylation may be required for GlcCer function in cellular membranes.

Another example is given by *Magnaporthe oryzae*, a distinct plant pathogenic fungus responsible for a devastating disease affecting rice crops worldwide [68–70]. *M*. *oryzae* produces a specialized structure named appressorium, which is necessary for host infection [71]. Recently, Liu and colleagues [51] systematically analyzed the changes of various metabolites during the stages in the formation of appressorium of *M*. *oryzae* and found that certain sphingolipids, including sphingosine, phytoneurosides, dihydroneurosine, and ceramide, were accumulating during appressorium formation. In fact, whereas ceramide content was extremely low in conidia, it increased drastically with the appressoria formation as early as 12 h and gradually decreased after 24 h [51]. Pharmacological inhibition of this ceramide synthesis by myriocin or fumonisin B1 impairs appressorium formation [51]. That ceramide is essential for appressoria was also shown by a recent study by Zhu and colleagues [72] in which deletion of 2 VASt domain containing proteins, *MoVAST1* or *MoVAST2*, results in low ceramide content and abnormal appressoria [72,73]. Furthermore, deletion of MoLag1, encoding for ceramide synthase, produces a mutant, Δ*Molag1*, with reduced hyphal growth and conidiation and loss of pathogenicity due to the lack of C19:2/C18 GlcCer, the major species of GlcCer produced by this fungus. Of note, *M*. *oryzae* also produces galactosylceramide (GalCer), and deletion of the gene responsible for its production, *MoCGT1*, causes serious defects in the cell development, appressorium formation, and infection [51]. Of note, in addition to providing fitness to fungal cells, fungal GlcCer may directly damage plant cells because when sprayed onto rice leaves, it damages them [23,74]. Taken together, these studies suggest that the production of this complex sphingolipid in fungi pathogenic to plants is of great significance to their virulence characteristics and it helps the fungus and damages the plant at the same time.

### 4. Regulatory mechanisms of sphingolipids biosynthesis in pathogenic fungi

Despite the fact that sphingolipids are essential lipids in most eukaryotes, the production process does create harmful metabolites [75]. Accordingly, the level of these metabolites must be precisely controlled to make sure their intracellular contents do not reach toxic level [42]. This intracellular level may be controlled biochemically by controlling the kinetic of the enzyme(s) responsible for their synthesis or/and by controlling the breakdown of those toxic sphingolipids, or/and by controlling their trafficking and movement within the cell [2]. However, how these control mechanisms are regulated is largely unknown.

In recent decades, small membrane-bound proteins were identified as central regulators of SPT activity in yeasts and animals. Orm1 and Orm2, 2 orosomucoid (ORM) proteins reported in *S*. *cerevisiae*, act as negative regulators of sphingolipid biosynthesis and are redundant from the functional perspective. The knockout of Orm1 and Orm2 can up-regulate level of PHS to roughly 5 times higher than wild-type and make mutant cells more responsive to exogenous sphingolipids [41,76]. Breslow and colleagues suggested that Orm1/2 directly binds Lcb1, Lcb2, and Tsc3 to produce the SPT complex in *S*. *cerevisiae* [41]. In the pathogenic fungus *M*. *oryzae*, only 1 Orm protein was identified. However, in contrast to *S*. *cerevisiae*, deletion of *MoORM1* did not cause any changes in sphingolipid levels [72]. This suggests that specific function(s) of a specific sphingolipid(s) cannot be extrapolated from studies performed in different fungi, but rather must be studied in that specific fungus.

Additional evidence of sphingolipid regulation is given by studies on the Ypk1 homolog Aga1 in the smut fungus *U*. *maydis*. Aga1 deletion mutant was affected in polarized growth and appressoria formation and showed defects in actin organization [77]. Interestingly, the phenotypes of Δ*aga1* in *U*. *maydis* were similar to sphingolipid biosynthesis-related gene loss mutants in *M*. *oryzae* and *F*. *graminearum*, suggesting that Aga1 may act as an upstream AGC kinase to regulate sphingolipid synthesis in this fungal plant pathogen. This hypothesis is supported by studies showing that in the Δ*Movast1* mutant, the sphingolipid synthesis was inhibited due to low activity of TORC2-Ypk1 [72,73], suggesting that, in *M*. *oryzae*, MoVast1 is a novel upstream regulator of the sphingolipid pathway.

### 5. The cross-talk between the sphingolipid pathway and other pathways involved in the regulation of fungal pathogenicity

Various infection strategies have been developed based on the long-term competition between pathogens and plants [77]. Appressoria are specialized infection structures produced by a variety of plant pathogenic fungi and are crucial to successfully invading their host plants [68]. Several pathogenic related signaling pathways, including cAMP-PKA, Pmk1-MAPK, TOR, autophagy, and endocytosis, have been verified to participate in this infection process in *M*. *oryzae*, *U*. *maydis*, and *F*. *graminearum* [78–81]. As revealed by numerous studies conducted over the past decade, these signaling pathways interact with one another and with the sphingolipid biosynthetic pathway in the regulation of pathogenicity by plant pathogenic fungi [82,83]. Interestingly, some of these pathways are conserved in nonpathogenic fungi. For example, in the yeast *S*. *cerevisiae*, Pkh1 and Pkh2, which act as crucial kinases that phosphorylate Pkc1 and activate PKC-CWI signaling, can be activated by DHS or PHS [84,85]. In *M*. *oryzae*, the PKC-CWI signaling regulates the infection progress by mediating the cell cycle of appressoria [86]. Loss of ceramide by deletion of MoLag1 led to a significantly reduced phosphorylation of Mps1. Interestingly, exogenous treatment with ceramide partially restored Mps1 phosphorylation in the *Molag1* mutant [51,87]. These results suggest that ceramide regulates the PKC-MAPK pathway and cell wall integrity in *M*. *oryzae*.

The endocytosis pathway is a conserved pathway that takes up macromolecules, PM, and extracellular material, thus relying on vesicle trafficking for nutritional absorption, cell polarity, and signaling transmission [88]. In pathogenic fungi, the endocytosis pathway plays crucial roles in virulence. The functions of sphingolipids in endocytosis have been recently revealed in several fungi [56,89,90]. Blocking the production of LCBs results in endocytosis defects, which can be compensated by the exogenous supplementation of LCBs [91].

Moreover, the distribution and trafficking of sphingolipids in the PM is controlled by the endocytosis pathway. Specific ABC proteins, members of the ATP-binding cassette, localized to the ER-PM contact site are controlling this trafficking in an endocytosis-dependent and endocytosis-independent manner (non-vesicular transport), playing a certain role in the outward translocation of sphingolipids, including ceramide, S1P, and GlcCer [92–94]. Studies have suggested that Aga1, a protein with similar functions to Ypk1 in *U*. *maydis*, may indirectly regulate sphingolipid production by regulating endocytosis. This indicates that the endocytosis pathway can help key sphingolipids to be delivered at different organelles for their function or for further synthesis. For example, ceramide is transported from the ER to the Golgi through endocytic vesicles for the synthesis of GlcCer [95]. Thus, in being components of endocytic vesicles, sphingolipids can participate in a variety of cellular processes regulating fungal fitness in the host.

Sphingolipids are also involved in the regulation of the autophagic process in plant pathogenic fungi. Autophagy is a highly conserved pathway, with the ultimate goal to specifically degrade intracellular components and to promote molecular recycling [73]. Inappropriate autophagy, and thus inappropriate recycling, significantly impairs cell growth and fitness in eukaryotes [73]. As indicated by the result of Muir and colleagues [96], the accumulation of intermediates of the sphingolipid pathway, such as the long-chain base 1-phosphate (LCBPs), can impair cell growth due to an improper autophagic response. This may lead to a further activation of sphingolipid production by TORC2-Ypk1 signaling, which is accompanied by an additional accumulation of toxic LCBPs metabolites [96].

In most plant pathogenic fungi, autophagy related proteins (ATGs) are virulence factors, and they regulate the pathogenic process. For example, all the key ATGs (e.g., Atg1, Atg4, Atg7, Atg8, Atg9, and Atg14) in *M*. *oryzae* are reported to regulate formation of appressoria. Deletion of these Atg proteins causes a drastically decrease of the turgor pressure of appressoria leading to the inability of the fungus to penetrate the plant host cuticle [97–100]. Liu and colleagues [51] recently suggested that this turgor pressure of appressoria requires ceramide, and when ceramide is lacking due to the dysfunction of ATGs, this pressure is lacking, the appressorium does not mature, and *M*. *oryzae* loses its infectivity [51]. On the other hand, excessive autophagy is also detrimental, as revealed by the study of Zhu and colleagues [72], in which deletion of lipid binding proteins in *M*. *oryzae* disturbs the activity of the TOR complex, resulting in excessive autophagy, thereby impacting the pathogenicity of rice blast fungus [72,73]. These results suggest that the autophagic process is tightly linked to the sphingolipid pathway.

### 6. Structural analysis of sphingolipid metabolizing enzymes in mammals and fungi

**Fig 3:**
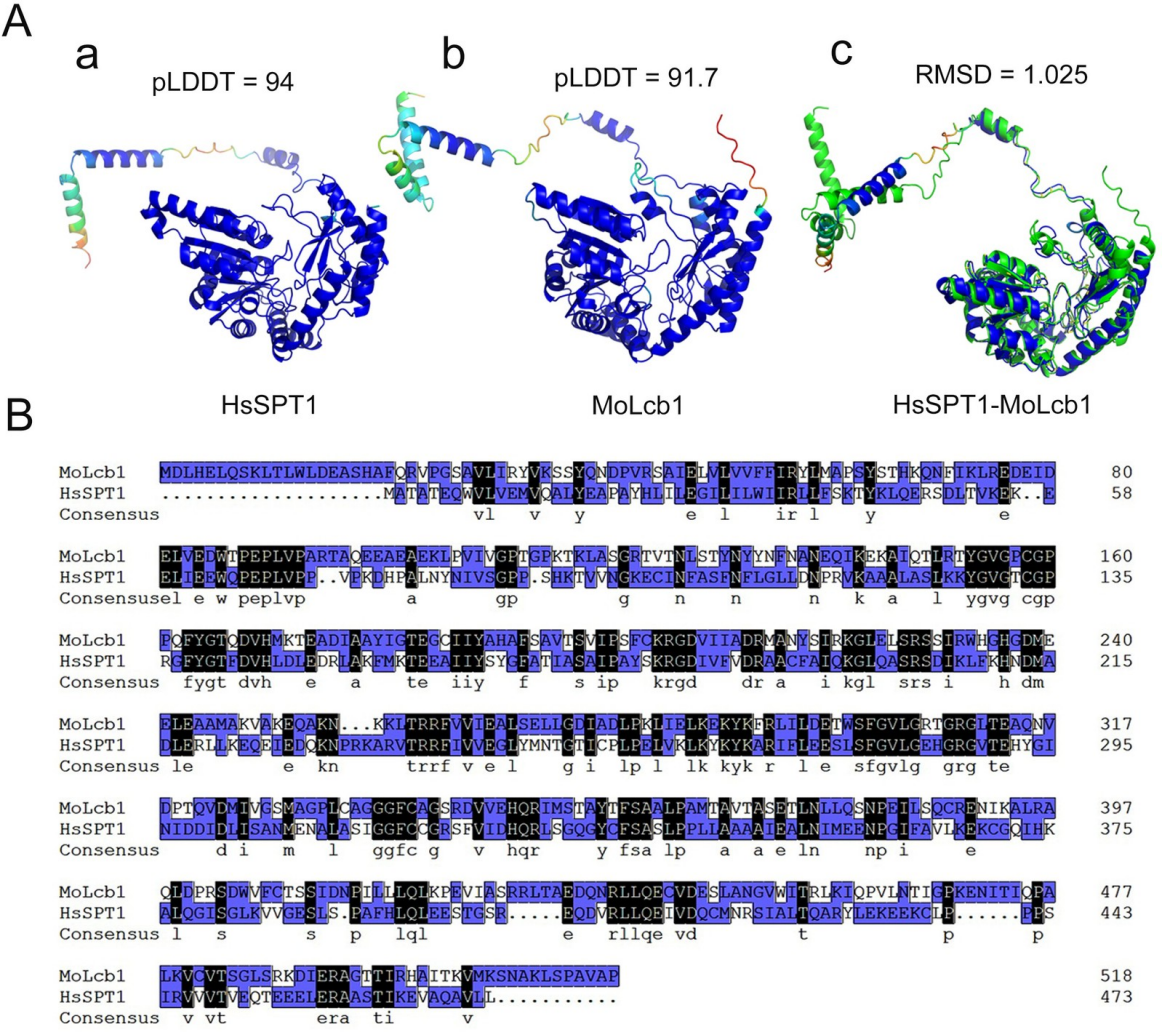
Structures of sphingolipid synthesis regulators between mammals and fungi. (A) (a) The structure of HsSPT1 was constructed using AlphaFold2. (b) 3D structure of MoLcb1 of *M*. *oryzae*. (c) The alignment structures of HsSPT1 (blue) and MoLcb1 (green). (B) Multiple sequence alignment of Spt1 proteins using MEGA 7.0 software. SPT, serine palmitoyltransferase.

The enzymatic structural data at atomic resolution provides tremendous potential for drug design and precise targeting therapy. However, thus far, we are not aware of any protein crystal structure that has been solved by conventional experimental methods related to an enzyme involved in the sphingolipid pathway. An accurate prediction of protein structure has become possible with the recent development of artificial intelligence. AlphaFold2, a deep machine learning algorithm developed by DeepMind for predicting protein structure based on a protein sequence, has notably advanced protein structure prediction [101]. In the AlphaFold Protein Structure Database (https://alphafold.ebi.ac.uk/), almost all protein structures have been predicted. In humans, the structure of sphingolipids synthesis key protein HsSPT1 was predicted with high confidence (pLDDT = 94). In the plant pathogenic fungus *M*. *oryzae*, the SPT MoLcb1 exhibits high similarity (RMSD = 1.025) with HsSPT1, even though the protein sequences only achieve 35.55% score of identity (Fig 3A and 3B). This indicates that the de novo synthesis system of sphingolipids has high structure conservation from animals to fungi. While these structures await experimental validation, computational drug design using molecular docking (e.g., DOCK6) can facilitate the early-stage drug discovery.

### 7. The identification of compounds targeting the sphingolipids pathway

In recent years, the differences in sphingolipid structure and in sphingolipid-metabolizing enzymes between mammals and fungi have been exploited for the research and development of new antifungals. Several compounds (e.g., myriocin, fumonisins, aureobasidin A, galbonolide, and acylhydrazones) targeting fungal sphingolipids biosynthesis-associated proteins have been developed over the past decades [62,102,103]. In addition, some of these compounds, such as myriocin that block SPT activity, the first step of the sphingolipid pathway, has been used as a useful tool to study the effect of the lack of sphingolipids in biological processes in mammalian, fungal, and plant cells [102,104]. Other compounds, such as fumonisins, targeting downstream enzymes of SPT, such as ceramide synthases, exhibit a broad-spectrum antifungal activity, and are synthesized by *Fusarium* species. They also target mammalian ceramide synthase. Perhaps, when the ceramide synthase crystal structure will be available, we can study how fumonisins binds to its target and envision the chemical synthesis of fungal-specific inhibitors. Aureobasidin A, a natural compound that inhibits Ipc1, a fungal-specific enzyme, has been reported as an exciting compound [62], but its cumbersome structure limits the production of better derivatives. He and colleagues [105] recently reported 3 herbicides (i.e., metazachlor, cafenstrole, and diallate) that are capable of inhibiting VLCFAs, exhibiting a broad-spectrum fungicidal activity against a variety of plant fungal pathogens (e.g., corn, wheat, and locusts) [105] (Table 2).

**Table 2:** Some sphingolipid inhibitors against phytopathogenic fungi.

Inhibitors	Target	Toxic		
		Animals	Fungi	Plants
Myriocin	SPTs	Improves glucose homeostasis and resolves hepatic steatosis [106]	Exhibit biological activity against yeast and plant pathogenic fungi [72,73]	Induce plants resistance to FB1 and AAL-toxin [107,108]
Fumonisins	Ceramide synthetase	Show high toxic to mammals	Exhibit biological activity against yeast and plant pathogenic fungi [109]	Impairs plant growth and development [109]
Aureobasidin A Galbonolide	IPC	Display low toxicity to mice [102]	High specific inhibit activity to fungi [60]	No phytotoxic [110]
BHBM D0	GlcCer	Show very low toxicity to mammals [111]	Show broad-spectrum antifungal activities with very high specificity [96]	Not reported
Metazachlor Cafenstrole Diallate	VLCFA elongase	Without toxicity to locusts [105].	New broad-spectrum fungicides [105]	No phytotoxic to maize and wheat [105]

IPC, inositol-phosphoryl ceramide; SPT, serine palmitoyltransferase; VLCFA, very long chain fatty acid.

Finally, acylhydrazones have emerged as promising antifungal agents affecting the fungal but not the mammalian sphingolipids. They have a broad-spectrum antifungal activity in vitro and in animal models of human fungal infections and a promising therapeutic window [62]. Research should be conducted to test whether these compounds will also be efficacious in protecting plants from pathogenic fungi.

## 8. Conclusions

Sphingolipids play crucial roles in a variety of biological processes, as they are essential components of the PM, they are involved in the transduction of different intracellular signals, and they play a key role in cell polarization, differentiation, as well as cell migration [23,32,112,113]. Several studies highlighted that sphingolipids and their intermediates are crucial to fungal pathogens for the establishment of the infection process. Compared with human fungal pathogens, studies on fungi affecting plants are still limited. However, as we learn more about the sphingolipid pathway in plants and fungi afflicting plants, we are discovering a common and unique feature of sphingolipids in the regulation of the pathogenic process. Perhaps, this uniqueness will teach us on how we can better protect our crops from these devastating fungal diseases. Importantly, studying the enormous variety of sphingolipids in plants could also help us to better understand how plants adapt to the changes in the environment, particularly in the adaptation to cold and warm temperature, leading to important insights on how we can better control the effect of climate change on crops and human health.

The identification of pharmacological inhibitors and the resolution of protein 3D structures will expedite the advance of novel technologies and novel therapeutic strategies to combat plant pathogenic diseases.
